# Towards Broadband High-Frequency Vibration Attenuation Using Notched Cross-Shaped Metamaterial

**DOI:** 10.3390/mi14020414

**Published:** 2023-02-09

**Authors:** Jin Guo, Rui Zhao, Yunbo Shi

**Affiliations:** Science and Technology on Electronic Test & Measurement Laboratory, School of Instrument and Electronics, North University of China, Taiyuan 030051, China

**Keywords:** plate-type metamaterial, broadband, vibration attenuation, bandgap

## Abstract

This paper reports a plate-type metamaterial designed by arranging unit cells with variable notched cross-sections in a periodical array for broadband high-frequency vibration attenuation in the range of 20 kHz~100 kHz. The dispersion relation and displacement field of the unit cell were calculated by simulation analysis, and the causes of the bandgap were analyzed. By studying the influence of critical structural parameters on the energy band structure, the corresponding structural parameters of a relatively wide bandgap were obtained. Finally, the plate-type metamaterial was designed by arranging unit cells with variable notched cross-sections in the periodical array, and the simulation results show that the vibration attenuation amplitude of the metamaterial can reach 99% in the frequency range of 20 kHz~100 kHz. After fabricating the designed plate-type metamaterial by 3D printing techniques, the characterization of plate-type metamaterial was investigated and the experiment results indicated that an 80% amplitude attenuation can be obtained for the suppression of vibration with the frequency of 20 kHz~100 kHz. The experimental results demonstrate that the periodic arrangement of multi-size cell structures can effectively widen the bandgap and have a vibration attenuation effect in the bandgap range, and the proposed plate-type metamaterial is promising for the vibration attenuation of highly precise equipment.

## 1. Introduction

High-frequency vibration with the range of 20 kHz~100 kHz often leads to lower stability and accuracy, especially for the applications of atomic force microscope tests and non-destructive testing [1]. In addition, high-frequency vibration is one of the causes of the failure of electronic components [2]. Carrier rockets, satellites, and aircraft often encounter high-frequency vibration shocks during flight, which can easily lead to the failure of the equipment’s electronic equipment. For example, the rocket sled test system is affected by the high-frequency vibration caused by the clearance between the slide rail and the rocket sled. Therefore, developing an efficient technique for broadband high-frequency vibration attenuation is of the utmost importance. However, porous materials and viscoelastic materials have the problems of narrow damping bands and less frequency selectivity, making it challenging to attenuate broadband high-frequency vibration effectively. In the last two decades, artificially engineered structures or metamaterials with unconventional mechanical properties have attracted extensive research interest. With the continuous research on electromagnetic metamaterials, the concepts of acoustic metamaterials and elastic metamaterials have been gradually proposed, and the manipulation, guidance and dissipation of acoustic and elastic waves have been realized. To date, acoustic metamaterials and elastic metamaterials have been used in a wide range of applications, from acoustic lens design [3], cloaking [4], and programmable metasurfaces [5], to diodes [6], logic-based acoustic switches [7] and nonreciprocal systems [8]. It is well known that metamaterials have filtering properties, where acoustic and elastic waves do not propagate freely in some frequency ranges, which are called band gaps. This property has attracted great interest in the field of vibration isolation. The band gap property of metamaterials is usually formed by the Bragg scattering mechanism and the local resonance mechanism. For Bragg scattering metamaterials, the wavelength of the band gap band is almost equal to the lattice constant of a single unit cell. For locally resonant metamaterials, it was first demonstrated by Liu et al. who fabricated three-dimensional phonon crystals consisting of a cubic array of coated spheres immersed in an epoxy resin matrix, which produced a subwavelength band gap that was mechanically tunable and independent of cell size, making acoustic metamaterials attractive for a wide range of vibration control applications [9]. Depending on the application, metamaterial structures can be designed as rods, plates, flex beams, inclusions, resonators, among others.

Plate-type metamaterial is an artificial composite structure with bandgap characteristics, which has demonstrated the existence of a complete bandgap to control broadband vibration effectively [10,11,12,13,14]. Various research achievements have exhibited high capability for the attenuation of vibration by plate-type metamaterials with soft/composite pillars, plates with double-side pillars, and tubular pillars [15,16,17,18,19,20,21,22]. The results have indicated that the ratio of the pillar thickness to the lattice period is the key parameter for the existence and the width of these complete bandgaps. Moreover, with respect to the bandgap broadening, many researchers have introduced local resonators with multi-degree-of-freedom vibration into the design of metamaterials [23,24]. Yan et al. widened the bandgap and controlled the in-plane wave propagation characteristics of two-dimensional functionally graded periodic grid structures by adding local resonators made of a spring oscillator [25]. Some researchers have designed some special structures to widen the bandgap of metamaterials, such as screw-type [26], sandwich-plate-type [27], honeycomb-type [28], and accordion-like-type [29] structures. In addition, many researchers have widened the bandgap by superimposing different bandgaps to form multiple bandgaps. Wen et al. studied a uniform string with periodically attached spring-mass resonators and found that coupled local resonance and Bragg phonon crystals can obtain a wide bandgap [30]. By conducting more studies on the local resonant band gap, the existing local resonant metamaterials with multiple band gaps have been classified into series mode and parallel mode. In the series mode, the local resonator oscillators can be directly connected and most of the multilevel series oscillators are attached to the substrate or structure surface in the form of adhesion for manufacturing convenience. For example, Peng et al. added secondary oscillators to primary oscillators to form a double-bandgap resonant structure [31]. Zhou et al. proposed a kind of multi-coaxial cylindrical inclusions and found that by periodically embedding multilayer coaxial inclusions, the band gap of a local resonant phononic crystal can be extended to multiple frequency ranges, realizing the series mode of embedded oscillators [32]. However, this form of oscillator is limited by the unit cell size, which affects the number and width of band gaps [33]. One way to increase the bandwidth of the transmission loss without increasing the mass is to reconfigure the mass arrangement of the resonators in the microstructure. Steven et al. analyzed a variety of membrane resonators with ring masses and found that a change in the uniformity of the center mass and ring mass distribution increases the total bandwidth of the local resonant transmission loss of the membrane resonator, while increasing the radius of the ring to approach the membrane radius decreases the total bandwidth of the local resonant transmission loss of the membrane resonator [34]. In contrast, when embedded parallel oscillators are adopted, the resonant frequencies of each oscillator can be designed and adjusted by the desired frequency band without periodic limitations [35]. Stein et al. found that the bandgap of metamaterials can be widened by introducing multiple parallel oscillators [36]. Tian et al. theoretically and experimentally investigated the elastic metamaterials with two parallel local resonators and effectively created two bandgaps of the bending wave [37]. Xiao et al. proposed a panel with multiple periodic arrays of attached resonators and achieved multiple locally resonant bandgaps [38,39]. Meng et al. established a theoretical model for joining multiple locally resonant bandgaps of two-phase composites by damping. Based on the model, two-phase locally resonant materials were optimized to obtain a super-wide bandgap [40]. Lu et al. designed a new type of metamaterial plate and analyzed its vibration characteristics by using the finite element method. They found that compared with regular shapes, irregular shapes of oscillators more easily open bending bandgaps [41]. Although the bandgap properties of the conventional periodic structures have been extensively investigated for their potential applications to vibration and sound isolation, few studies report metamaterials that use multi-size unit cell combination periodic arrangement and the vibration attenuation technique towards the frequency band range of 20 kHz~100 kHz by using plate-type metamaterials.

In this work, considering that irregular shapes of oscillators more easily open bandgaps and the various forms of plate-type metamaterials, we designed a plate-type elastic metamaterial with a periodic notched cross shape to attenuate the broadband high-frequency vibration within the range of 20 kHz~100 kHz. The dispersion relation and displacement field of the unit cell were calculated by simulation analysis, and the causes of the bandgap were analyzed. By studying the influence of critical structural parameters on the energy band structure, the corresponding structural parameters of a relatively wide bandgap were obtained. Finally, the plate-type metamaterial was designed by arranging unit cells with variable notched cross-sections in the periodical array, and the simulation results show that the vibration attenuation amplitude of the metamaterial can reach 99% in the frequency range of 20 kHz~100 kHz. After fabricating the designed plate-type metamaterial by 3D printing techniques, the characterization of plate-type metamaterial was investigated and the experiment results indicated that an 80% amplitude attenuation can be obtained for the suppression of vibration with the frequency of 20 kHz~100 kHz by using the fabricated plate-type metamaterial.

## 2. Design and Simulations

**Figure 1:**
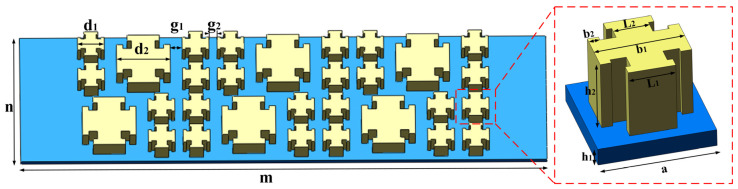
The schematic of the metamaterial and the corresponding unit cell, which comprises a square resinic substrate and a notched cross-shaped steel mass block.

The schematic diagram of the proposed plate-type metamaterial is shown in Figure 1, which comprises a square resinic substrate and a notched cross-shaped steel mass block. The corresponding Young’s moduli are 2.5 GPa and 200 GPa, and the Poisson ratios are 0.41 and 0.27, respectively. To enhance the bandgap property, the specimen was constructed by arranging unit cells with variable notched cross-sections in the periodical array. The overall length and the width of the plate-type metamaterial are represented by m and n, the side length of the small-sized metal structure is d_1_, the side length of the large-sized metal structure is d_2_, the gap between large-sized and small-sized metal structures is g_1_, and the gap between two small-sized metal structures is g_2_.

**Figure 2:**
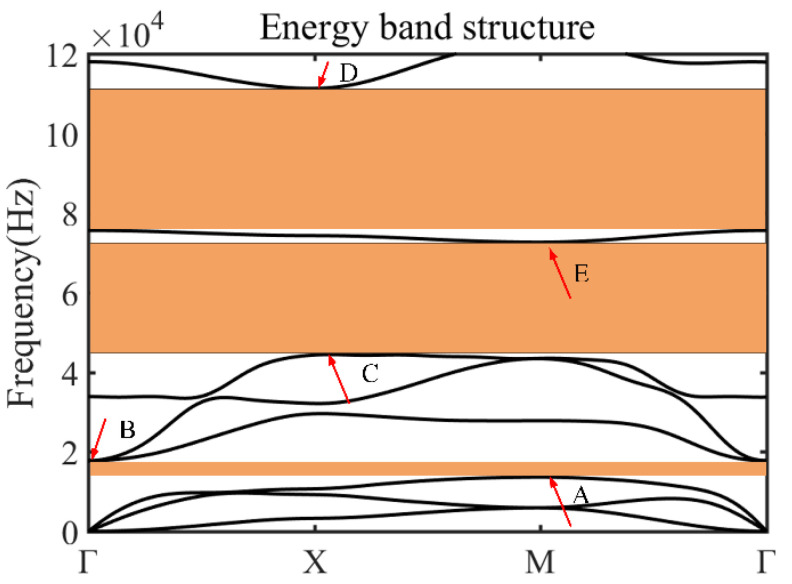
The schematic diagram of energy band structure for the designed unit cell. The parameters used are as follows: a = 7 mm, h_1_ = 1.5 mm, h_2_ = 3 mm, b_1_ = 3 mm, L_1_ = 2.6 mm, L_2_ = 2 mm, b_2_ = 0.6 mm.

**Figure 3:**
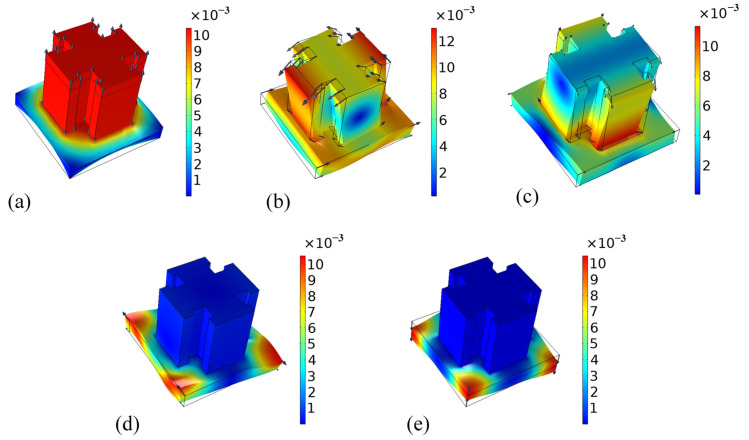
The displacement vector fields of the extracted intrinsic points in the energy band structure. (**a**) Mode A; (**b**) Mode B; (**c**) Mode C; (**d**) Mode D and (**e**) Mode E.

The plate-type metamaterial is designed by combining the Bragg-scattering and locally resonant bandgap mechanism [42,43,44,45]. Based on the Floquet–Bloch wave theory [46], the geometric parameters are calculated, as sketched in Figure 1. In this study, all the numerical calculations were carried out using the commercial software COMSOL Multiphysics 5.5. The parameters of the unit cell are described as follows: a = 7 mm, h_1_ = 1.5 mm, h_2_ = 3 mm, b_1_ = 3 mm, L_1_ = 2.6 mm, L_2_ = 2 mm, b_2_ = 0.6 mm. Figure 2 shows the energy band structure of the unit cell in the frequency range of 0 Hz~100 kHz, where five intrinsic points were extracted to show the vector displacement field of the unit cell corresponding to Figure 3. For the case of mode A, the coupling between the antisymmetric Lamb wave mode of the substrate and the stretch vibration of the mass block resulted in the generation of the lower edge of the first complete bandgap. The energy was concentrated within the notched cross structure, and then generated a high-frequency bandgap. For the case of mode B, it exhibited Lamb wave propagation with energy propagating in the substrate, which indicated that the bandgap had completely disappeared and elastic waves began to propagate along the substrate. Mode C, corresponding to the lower edge of the second complete bandgap, is represented by a slight torsional vibration of the mass block and a slight bending motion within the substrate surface. Mode D at the upper edge exhibited the state of longitudinal vibration along the *z*-axis of the substrate. At this point, the mass block remained stationary, and the Lamb wave propagated along the substrate. The flat band shown by mode E in the bandgap was formed by the vibration of the substrate edge, which indicated that there was no effect on the formation of the wide bandgap. All these simulations demonstrated that the formation of the first complete bandgap could be attributed to the strong coupling between the vibration of the notched cross structure and the substrate Lamb waves, and the lattice constant a, the substrate thickness h_1_, the metal thickness h_2_, and b_1_ had significant effects on the coupling strength. The high-frequency broadband-gap composed of the second and third complete bandgaps is generated by Bragg scattering.

**Figure 4:**
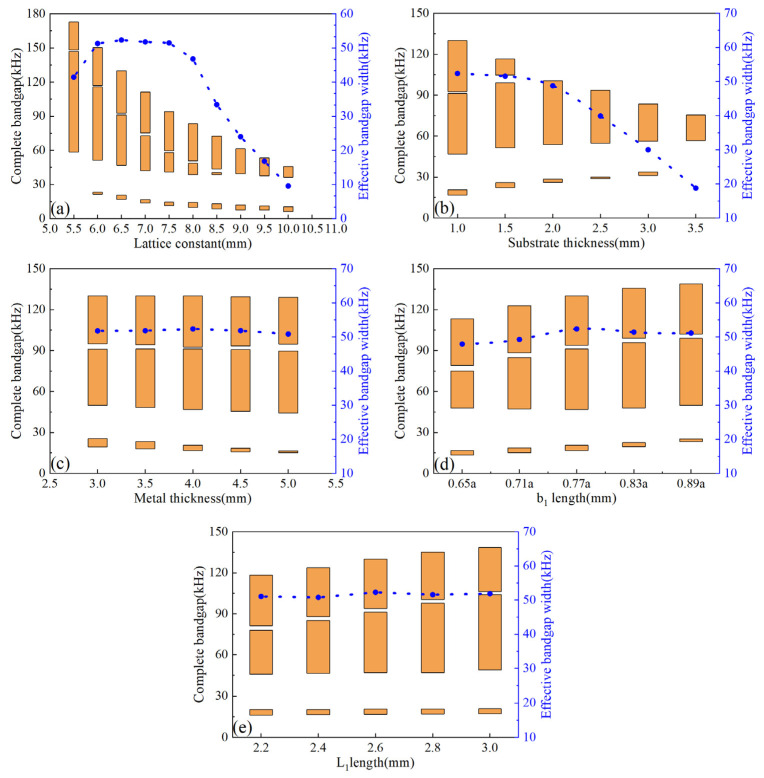
The change in the complete bandgap and the effective bandgap width versus (**a**) the lattice constant a of unit cell, (**b**) the substrate thickness h_1_, (**c**) the metal thickness h_2_, (**d**) the length of b_1_, and (**e**) the length of L_1_.

To broaden the response range of frequency, the simulations were performed by varying from the parameters of a, h_1_, h_2_, b_1_, and L_1_. The dispersion responses are simulated for the lattice constant from 5.5 mm to 10 mm, while keeping h_1_, h_2_, b_1_, and L_1_ constant. Figure 4a shows the variation trends of the complete bandgap and the effective bandgap width with the increase in the lattice constant a. We can observe that both the upper and lower edges of the bandgap moved to the low-frequency domain simultaneously, and the upper boundary dropped much more quickly. This result was caused by the decrease in the coupling area. It is worth noting that there were three bandgap domains when the lattice constant was selected within the range of 6 mm~8.5 mm. However, when the lattice constant was outside the range, the bandgap domains decreased from three domains to two domains and the bandgap width became narrower. For Figure 4b, keeping a, h_2_, b_1_, and L_1_ constant, with the substrate thickness h_1_ increasing, the domains between the upper and lower edge of the bandgap narrowed down, and the three bandgap domains overlapped completely. These phenomena can be contributed to the weaker coupling between the notched cross structure and the substrate. The simulations of the metal thickness h_2_ that affected the bandgap are shown in Figure 4c. It can be seen that the variation in metal thickness only affected the bandgap width of the low-frequency domain. Figure 4d shows the upper and lower edges of the bandgap moved to the high-frequency domain, and the overall bandgap width increases when the length of b_1_ gradually becomes closer to the lattice constant. It is worth pointing out that the bandgap domains of low frequency gradually narrowed down. Figure 4e shows that the variation in L_1_ length affected the bandgap width of the high-frequency domain, and the bandgap center frequency increased with the increase in L_1_ length but has no significant effect on the effective bandgap width.

**Figure 5:**
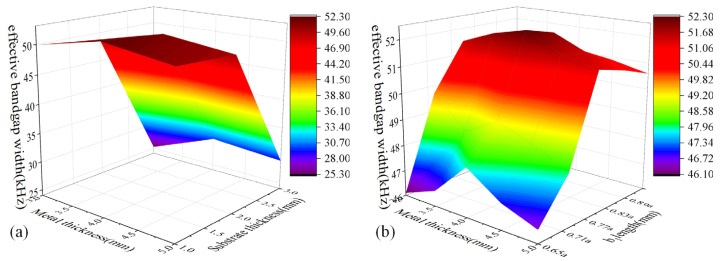
(**a**) The effect of h_1_ and h_2_ on effective bandgap width; (**b**) the effect of h_1_ and b_1_ on effective bandgap width.

**Figure 6:**
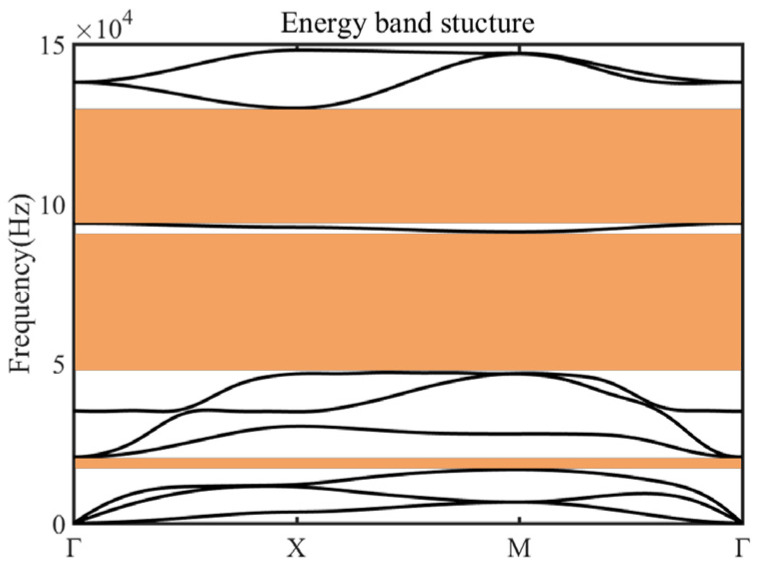
The schematic diagram of the energy band structure for the 6.5 mm unit cell. The parameters used are as follows: a = 6.5 mm, h_1_ = 1 mm, h_2_ = 4 mm, b_1_ = 2.5 mm, L_1_ = 2.6 mm, L_2_ = 2 mm, b_2_ = 0.6 mm.

**Figure 7:**
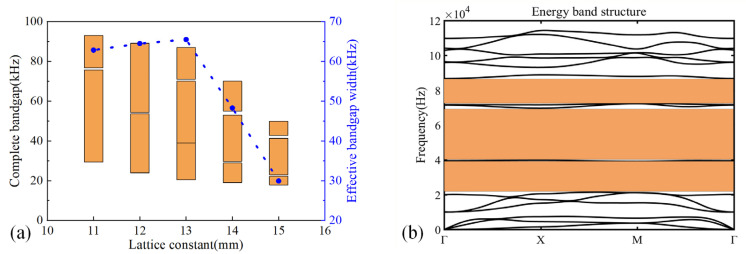
(**a**) Bandgap characteristics of the unit cell with different lattice constants when h_1_ = 1 mm, h_2_ = 4 mm, and b_1_ = 0.77a. (**b**) The energy band structure for the 13 mm unit cell. The parameters used are as follows: a = 13 mm, h_1_ = 1 mm, h_2_ = 4 mm, b_1_ = 10 mm, L_1_ = 6 mm, L_2_ = 4.4 mm, b_2_ = 1.2 mm.

The influence of h_1_ and h_2_ on effective bandgap width is shown in Figure 5a. It can be found that the effective bandgap width decreases with the increase in h_1_ within the range of 1 mm~3 mm, especially for the case from 2 mm to 3 mm, the effective bandgap width decreased rapidly and the change range was higher than 20 kHz. The effective bandgap width increased firstly and then decreased with the increase in h_2_, but the change range varied within the range of 5 kHz. Figure 5b exhibits the influence of h_2_ and b_1_ on the effective bandgap width. We can observe that the effective bandgap width increased firstly and then decreased with h_2_ and b_1_ increasing, but a significant effect was obtained for the case of b_1_. Thus, the coupling between parameters has no significant effect on the effective bandgap width [36]. Therefore, the parameters of the unit cell were optimized as follows: a = 6.5 mm, h_1_ = 1 mm, h_2_ = 4 mm, b_1_ = 2.5 mm, L_1_ = 2.6 mm, L_2_ = 2 mm, b_2_ = 0.6 mm. The energy band structure of the unit cell is shown in Figure 6. The band structure exhibited two bandgaps with the range of 16.8 kHz~20.63 kHz and 46.8 kHz~130 kHz, but there was a passband from 20.63 kHz to 46.8 kHz in the target frequency domain. To achieve the full bandgap of the target frequency domain, the plate metamaterial was designed by arranging unit cells with variable notched cross-sections in the periodical array. Figure 7a shows the bandgap characteristics of the unit cell with different lattice constants when h_1_ = 1 mm, h_2_ = 4 mm, and b_1_ = 0.77a. It is obvious that a bandgap with the range of 20.63 kHz~46.8 kHz is generated when a is equal to 13 mm, where the widest effective bandgap width can be obtained simultaneously. The parameters of the bigger size unit cell were obtained as follows: a = 13 mm, h_1_ = 1 mm, h_2_ = 4 mm, b_1_ = 10 mm, L_1_ = 6 mm, L_2_ = 4.4 mm, b_2_ = 1.2 mm. This unit cell’s energy band structure is shown in Figure 7b.

**Figure 8:**
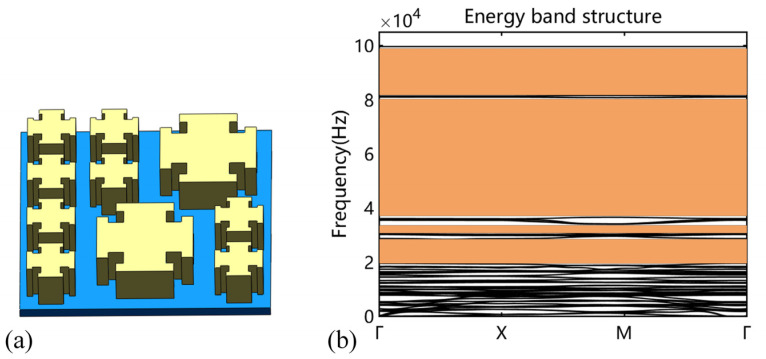
(**a**) The schematic diagram of the larger representative unit cell, including eight small blocks and two large ones; (**b**) the energy band structure of the larger representative unit cell.

**Figure 9:**
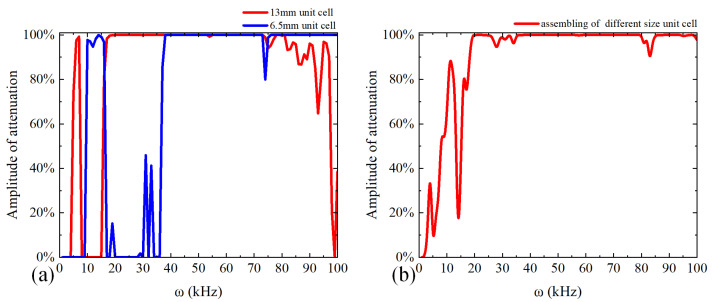
The velocity frequency response curve of the proposed plate-type elastic metamaterial with the frequencies of 0~100 kHz. (**a**) The plate metamaterial consisting of 6.5 mm unit cell and 13 mm unit cell, where the blue velocity frequency response curve represents 6.5 mm unit cell and the red velocity frequency response curve represents 13 mm unit cell; (**b**) the plate metamaterial which assembled the different size unit cell.

Figure 8a shows the larger representative unit cell, including eight small blocks and two large ones, whose energy band structure is shown in Figure 8b. We can observe that the band structure exhibited four bandgaps with the range of 19 kHz~28.6 kHz, 31.5 kHz~34 kHz, 36.8 kHz~80.7 kHz, and 81.5 kHz~99.6 kHz, which indicates that the proposed larger representative unit cell realizes the full coverage of the bandgap in the target frequency domain. The velocity frequency response curves of plate metamaterials consisting of a 6.5 mm unit cell and a 13 mm unit cell are described in Figure 9a. It is observed that the vibration signal for the plate metamaterial consisting of a 6.5 mm unit cell displayed a large amplitude attenuation in the 40 kHz~100 kHz range, and the plate metamaterial consisting of a 13 mm unit cell showed remarkable amplitude attenuation in the range of 20 kHz~80 kHz. These frequency ranges are consistent with bandgaps predicted by the energy band structure. Figure 9b shows the velocity frequency response curve of the plate metamaterial which assembled different size unit cells. The simulation result indicated that the vibration signal amplitude attenuation was about 99% with the frequency of 20 kHz to 100 kHz, which combined the bandgap of the single size unit cell and achieved the attenuation of vibration signals in the target frequency domain.

## 3. Experimental Results

**Figure 10:**
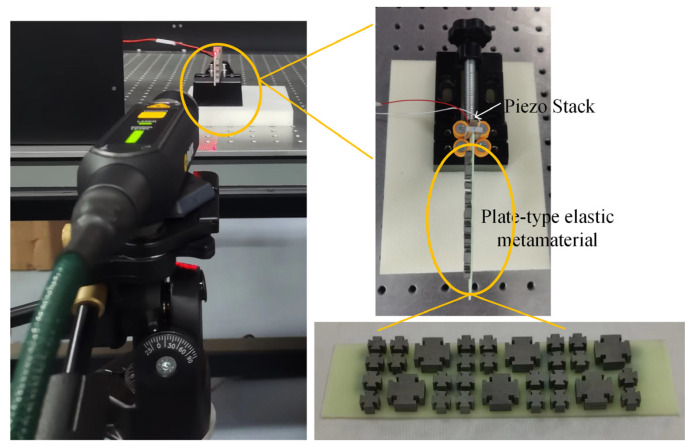
The setup of experimental measurement and the plate-type elastic metamaterial sample diagram.

**Figure 11:**
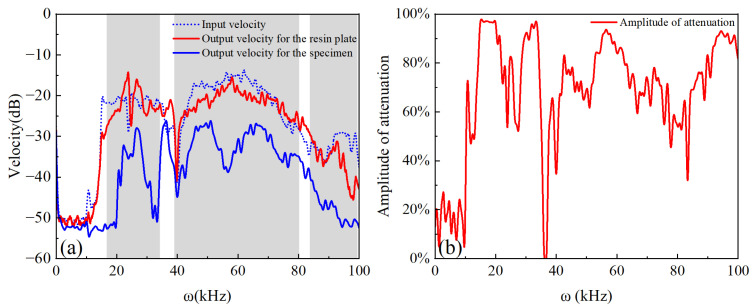
(**a**) The comparison of the input vibration velocity signal and output vibration velocity signal for the resin plate and the specimen within the range of the response frequency band, where the blue dashed line represents the input vibration velocity signal, the blue solid line represents the sample output vibration velocity signal and the red solid line represents the resin plate output vibration velocity signal; (**b**) the vibration signal amplitude attenuation for the specimen with the frequencies of 0~100 kHz.

The plate-type elastic metamaterial was fabricated by 3D printing techniques, as shown in the enlarged photograph in Figure 10, where high-toughness resin 8220 was used as the plate-type substrate, and stainless steel 316L was used for metal structures with the accuracy of ±0.15/100 mm. The vibration attenuation of the plate-type metamaterial was characterized by mechanical wave transmission experiments. The piezoelectric ceramic stack (NAC2013-H08, Noliac Inc., Albuquerque, NM, USA) was attached on one side of an aluminum sheet, while the metamaterial was fixed on the other side. A mechanical clamp was used to immobilize the aluminum sheet on an optical platform, and the specimen was parallel with the optical platform. During the experiments, a 10 V sine wave sweep signal with the frequency of 0 Hz~100 kHz generated by a signal generator was introduced into the piezoelectric ceramic stack to convert into a vibration signal, and the generated vibration signal would propagate on the specimen. The vibration velocity signal at the test point of the specimen was measured by the laser Doppler vibrometer (HSV-E-100, Polytec Inc., Karlsruhe, Germany). To further establish that the vibration attenuation is caused by the bandgap of the plate-type metamaterial, a comparison experiment was conducted, replacing the plate-type metamaterial with resin plate and with other conditions constant, and the results are shown in Figure 11. Figure 11a shows the comparison of the input and output vibration velocity signal for the resin plate and the output vibration velocity signal for the specimen, in which we can observe that these three signals have the same trend. The output signal for the resin plate has a significant transmission with respect to the input signal. However, for the shadow region in Figure 11a, it can be found that the output signal of the sample has significant attenuation relative to the input signal, which basically coincides with the theoretical simulation in Figure 8b. These phenomena show that the bandgap characteristics of plate-type metamaterial play an important role in attenuating vibration signals. Figure 11b shows the vibration signal attenuation level in the frequency range of 0 Hz~100 kHz. It can be found that the vibration signal at 20 kHz~100 kHz was attenuated with an amplitude greater than 80%. These experimental results indicated that there was a similar trend compared with the simulation results. When the vibration signal is in the passband range, the frequency response is most significant in the range of 35.9 kHz~36.9 kHz. Meanwhile, the experimental result has a 19% error range in the attenuation amplitude compared with the theoretical simulation. We believe that these phenomena can be attributed to the parameter errors of the fabricated specimen.

## 4. Conclusions

In this paper, a plate-type metamaterial is proposed to attenuate the broadband high-frequency vibration. The specimen was arranged by the construction of unit cells with variable notched cross-sections in the periodical array. The dispersion relation and displacement field of the unit cell were calculated by simulation analysis, and the causes of the bandgap were analyzed. By studying the influence of critical structural parameters on the energy band structure, the corresponding structural parameters of a relatively wide bandgap were obtained. Finally, the plate-type metamaterial was designed by arranging unit cells with variable notched cross-sections in the periodical array, and the simulation results show that the vibration attenuation amplitude of the metamaterial can reach 99% in the frequency range of 20 kHz~100 kHz. After optimizing the parameters of the unit cells, the specimen was fabricated by 3D printing techniques and its vibration attenuation characteristics with broadband high frequency were experimentally investigated. The experimental results demonstrate that the vibration signal within the frequencies of 20 kHz~100 kHz was attenuated with an amplitude greater than 80%. These results suggest that the periodic arrangement of multi-size cell structures can effectively widen the bandgap and have a vibration attenuation effect in the bandgap range. The plate-type metamaterial provides a promising method for the attenuation of high-frequency vibration, which can be used in the anti-vibration fields of high precision measuring equipment.

## Data Availability

The data that support the findings of this study are available from the corresponding authors upon reasonable request.
